# End-of-Life Care Factors Predict Affective Sequences in Older Adults’ Final Memories of Spousal Loss

**DOI:** 10.1093/geroni/igab046.3386

**Published:** 2021-12-17

**Authors:** Emily Mroz, Nerea Anaya-Dominguez, Susan Bluck

**Affiliations:** 1 Yale University, New Haven, Connecticut, United States; 2 University of Florida- Life Story Lab, Gainesville, Florida, United States; 3 University of Florida, University of Florida, Florida, United States

## Abstract

Memories from the dying days of a deceased spouse are vividly recalled and can guide grief adjustment in older adulthood (Mroz & Bluck, 2018). End-of-life factors (e.g., place of death, quality of death) likely impact the nature of recall of such memories over time. Intersecting psychology and palliative care perspectives, the current study employs mixed-methods to examine relations between end-of-life care factors and affective sequences in older adults’ final memories of spousal loss. Fifty-three participants (Mage = 81.59; M = 6.81 years since loss) completed a Final Memory Interview, provided place of spousal death (in hospital, outside of hospital), and completed the Good Death Inventory (GDI; Miyashita et al., 2008). GDI responses were organized into four quality of death categories. Final memories were reliably content analyzed for affective sequences (i.e., positive and negative affect themes; interrater agreements > .70): redemption (bad mitigated by good, McAdams 1999), contamination (good spoiled by bad; McAdams, 1998), positive stability, and negative stability. Loss of a spouse in hospital, compared to outside of hospital, related to narrating final memories with contamination, F = 4.05, p < .05. Quality of death predicted narration of final memories with positive affective sequences: lower reported comforting environment related to redemption (t = -3.05; p < .01) and higher reported appropriate medical care related to positive stability (t = 2.60; p < .05) in memories. As healthcare provision continues to adjust to improve end-of-life circumstances across care environments, the impact of circumstances on close others should factor into initiative development.

